# Supplementary material for: Building Trust and Awareness to Increase AZ Native Nation Participation in COVID-19 Vaccines
Source: Int J Environ Res Public Health. 2022 Dec 20;20(1):31. doi: 10.3390/ijerph20010031 (PMC9819330; doi:10.3390/ijerph20010031)
Supplement: Supplementary file 1 [file ijerph-20-00031-s001.zip › Handout S10.pdf]

GRIEF DURING COVID-19

Many families have been affected by COVID-19 through loss of loved ones and from sudden, unexpected changes.

These changes can be difficult because we care for our community and value the health of our family, elders, and children.

Things to know about grief:

- **Grief is a normal response** to loss and is not only for the death of a loved one. Other types of loss, such as losing a house or job, may cause grief as well.
- **Grief is personal** and there is no right or wrong way to grieve for a loss.
- **Grief has no timeline.**

Grief can show itself in different ways:

- Shock, disbelief, or denial
- Loss of sleep and appetite
- Periods of sadness or anxiety
- Anger or distress

Children and adolescents may show grief differently than adults. Promote healthy coping and acceptance:

- Ask questions to understand their emotional state.
- Allow them time to talk and express their feelings.
- Take care of yourself and model coping strategies for your child.

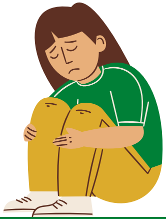

Resources: Centers for Disease Control & Prevention, Johns Hopkins Center for American Indian Health

COPING & HELP DURING COVID-19

Ways to relieve stress and cope with feelings of grief after a loss:

APACHE
BEHAVIORAL HEALTH SERVICES

Connect with your community

- Take part in sacred practices.
- Call family and friends to share feelings and experiences.
- Ask family and friends to share stories and pictures.
- Prayer and spiritual services can help - join virtually, by phone, or while physically distancing.
- Find a way to laugh every day.

Ask for help from others

- Take things day by day, some days may be harder than others.
- Connect with Mother Earth; go outside and enjoy nature, gather herbs and medicine.
- Seek support from friends, spiritual leaders, and over the phone services.

Resources

Apache Behavioral Health Services is available to you for emotional, social, and spiritual support. All calls are confidential.

Apache Behavioral Health 24/7 Helpline

- (928) 338-4811

Whiteriver Indian Hospital

- (928) 338-4911

24/7

GREAT SEAL
WHITE MOUNTAIN APACHE TRIBE

NORTHERN ARIZONA UNIVERSITY | Center for Health Equity Research

Funded by NIH/NIMHD U54MD012388
