# Supplementary material for: Building Trust and Awareness to Increase AZ Native Nation Participation in COVID-19 Vaccines
Source: Int J Environ Res Public Health. 2022 Dec 20;20(1):31. doi: 10.3390/ijerph20010031 (PMC9819330; doi:10.3390/ijerph20010031)
Supplement: Supplementary file 1 [file ijerph-20-00031-s001.zip › Handout S7.pdf]

COVID-19 VACCINE INFORMATION

WHAT IS THE COVID-19 VACCINE?

- The COVID-19 vaccine teaches your immune system to recognize and fight the virus that causes COVID-19.
- It creates a protein that builds antibodies to protect us from COVID-19.
- The COVID-19 vaccine DOES NOT contain the COVID-19 virus and it DOES NOT affect or interact with your DNA in any way.

Whiteriver Indian Hospital:
(928) 338-5549

ARE THE VACCINES SAFE?

- The development of COVID-19 vaccines in a short amount of time was possible because of a global community of scientists working together.
- Clinical trials show that the benefits of taking the vaccine outweigh the health risks of COVID-19.
- The vaccine is safe for people of all racial and ethnic groups. People with chronic conditions were also included in trials.
- Every study, every phase, and every trial for the vaccine was reviewed by the U.S Food and Drug Administration (FDA) and a safety board.
- The FDA has granted emergency use authorization for the Moderna and Johnson and Johnson COVID-19 vaccines, which are safe and effective.
- **Vaccine Update:** The FDA has granted full approval for the Pfizer-BioNTech COVID-19 Vaccine.

HOW CAN YOU BENEFIT FROM THE VACCINE?

- Will lessen infectious time for fully vaccinated people than unvaccinated people from the Delta variant.
- Will provide strong protection against serious illness from the Delta variant.
- Will keep you from getting really sick from COVID-19.
- Will be a safe way to build protection for yourself, family, & community.
- Will help everyone get back to their daily routines.
- Will be an important tool to help stop the pandemic.
- Will make sure the vaccines and/or treatments will work for as many people as possible.

Those who are immunocompromised can receive a booster shot of the COVID-19 vaccine at least 28 days after your second dose of Moderna or Pfizer.
COVID-19 booster shots for the general population will be available this fall.

Funded by NIH/NIMHD U54MD012388

Resources: Centers for Disease Control and Prevention, Johns Hopkins Center for American Indian Health

NORTHERN ARIZONA UNIVERSITY
Center for Health Equity Research

COVID-19 Vaccines Available

Pfizer-BioNTech

95% Effective

2 doses

21 days apart

Recommended for people 12 years and older*

Moderna

94% Effective

2 doses

28 days apart

Recommended for people 18 years and older*

Johnson and Johnson

85% Effective

1 dose

Recommended for people 18 years and older*

After you receive a dose of the the COVID-19 vaccine, you may experience a few side effects. These side effects are normal and may last a few days.*

**if you experience symptoms longer than 2 days, please contact your healthcare provider*

In the arm where you got the vaccine:

- Ache
- Swelling
- Redness

Throughout the rest of your body:

- Chills
- Tiredness
- Headaches

TIPS

To reduce pain or discomfort where you got the vaccine:

1. Apply a clean, cool, wet washcloth over the area
2. Use or exercise your arm

To reduce discomfort from fever:

1. Drink plenty of fluids and dress lightly
2. Talk to a healthcare provider about taking any over-the-counter pain medication

REMINDER

It takes time for your body to build protection after any vaccination. The COVID-19 vaccines require 1-2 weeks to provide protection.

****Vaccination is recommended for people who are pregnant, breastfeeding, and trying to get pregnant now or in the future.***

The White Mountain Apache Tribe is conducting COVID-19 Vaccine Clinics at local high schools for Native American/Alaskan Natives who are 12 years and older (Pfizer), 18 years and older (Moderna/Johnson & Johnson), and who normally receive healthcare at Whiteriver Service Unit.

Schedule an appointment at the hospital by calling (928) 338-5549

For questions about COVID-19 call the CHRs at (928) 338-4822
