# Supplementary material for: Building Trust and Awareness to Increase AZ Native Nation Participation in COVID-19 Vaccines
Source: Int J Environ Res Public Health. 2022 Dec 20;20(1):31. doi: 10.3390/ijerph20010031 (PMC9819330; doi:10.3390/ijerph20010031)
Supplement: Supplementary file 1 [file ijerph-20-00031-s001.zip › Handout S8.pdf]

COVID-19 Vaccine Frequently Asked Questions (FAQ)

FAQ: Will the vaccine give me COVID-19?

No. The vaccine does not contain the virus, it makes a protein like the one found in COVID-19. Our body sees this protein and builds antibodies to protect us. The vaccines are effective at preventing severe illness from COVID-19 and its variants.

FAQ: How long until the vaccine works? How long will it last?

It takes two weeks after your second dose of the Pfizer or Moderna vaccine, or the single dose for the Johnson & Johnson vaccine, to build protection against the virus and be fully vaccinated. The immune response from the first set of vaccines is likely to weaken over time, but we do know that getting the COVID-19 vaccine is a safer choice than not getting it. Booster shots will be available to the general public this fall.

FAQ: Do I need to wear a mask and physically distance after being vaccinated?

Yes. Continue wearing a mask and physically distancing when indoors and at crowded outdoor spaces, and where required by laws, rules, and regulations, including local business and workplace guidance.

FAQ: Does the vaccine protect against variants of the virus, like the Delta variant?

Yes. Infections happen in only a small proportion of people who are vaccinated, even with the Delta variant. Some "breakthrough infections," for those who have been vaccinated but still get the virus, are expected but are rare. Fully vaccinated people who are infected with the Delta variant can still spread the virus to others.

FAQ: Who can get vaccinated?

Everyone 12 years and older can get a COVID-19 vaccine to help protect against COVID-19. The vaccine is safe for people with chronic or underlying medical conditions and who are at an increased risk for severe illness from COVID-19.

FAQ: Why can't children under the age of 12 get vaccinated?

COVID-19 vaccines are not currently authorized for children between the ages of 2 and 11. Children can still be infected with, get sick from, and spread the virus. They should continue to wear a mask in public spaces and around people they don't live with to protect themselves.

FAQ: Can I get a COVID-19 vaccine if I am pregnant? What if I am breastfeeding? What if I want to get pregnant in the future?

Yes. Vaccination is recommended for people who are pregnant, breastfeeding, and trying to get pregnant now or in the future. Pregnant and recently pregnant people are more likely to get severely ill with COVID-19 compared to non-pregnant people, and getting a vaccine can protect you from severe illness.

Resources: Centers for Disease Control and Prevention, Mayo Clinic, Johns Hopkins Center for American Indian Health

Funded by NIH/NIMHD U54MD012388

NORTHERN ARIZONA UNIVERSITY
Center for Health Equity Research

GREAT SEAL WHITE MOUNTAIN APACHE TRIBE

Common COVID-19 Vaccine Myths

Myth: The side effects of the COVID-19 vaccine are dangerous.

Fact: You may have some mild side effects after vaccination, like pain, redness, and swelling in the arm where you received the shot, as well as tiredness, headache, and nausea. These are signs that your body is building protection. These side effects could affect your ability to do daily activities, but they are not dangerous and should go away in a few days. Do not let the side effects of the first dose keep you from getting your second, it is important to get fully vaccinated.

Myth: The COVID-19 vaccine has some sort of microchip that will track me.

Fact: There is no microchip, magnet, or any kind of technology similar to that in any of the COVID-19 vaccines. It is not possible to gather data and track people through the COVID-19 vaccines. This is a myth that was taken out of context and spread on social media.

Myth: Researchers rushed the vaccine. How can I trust it?

Fact: Different kinds of coronavirus and mRNA vaccines have been studied for decades. Researchers did NOT skip any steps while making the vaccines. They worked on different steps at the same time and had a lot of funding to get the vaccine out quicker than others. Native communities have suffered historical traumas and are in a healing mode, coming together to keep our community safe. Many people have lost their lives due to a lack of a vaccine during the pandemic. Tribal governments are working hard to keep their people safe.

Myth: If I have already had COVID-19 and recovered, I do not need a COVID-19 vaccine.

Fact: It is possible—although rare—that you could be infected with the virus again or variants of the virus. Experts do not yet know how long you are protected from getting sick again after recovering from COVID-19. The vaccines give protection against these variants and severe illness.

Myth: I have allergies, so I can't get the COVID-19 vaccine.

Fact: NONE of the COVID-19 vaccines contain eggs, preservatives, or latex. People with a history of severe allergic reactions not related to vaccines—such as food, pet, environmental allergies—should still get vaccinated. If you are allergic to other types of vaccines, ask a healthcare provider if you should get a COVID-19 vaccine.

The White Mountain Apache Tribe is conducting COVID-19 Vaccine Clinics at local high schools for Native American/Alaskan Natives who are 12 years and older (Pfizer), 18 years and older (Moderna/Johnson & Johnson), and who normally receive healthcare at Whiteriver Service Unit.

Schedule an appointment at the hospital by calling (928) 338-5549
For questions about COVID-19 call the CHRs at (928) 338-4822
