# Supplementary material for: Building Trust and Awareness to Increase AZ Native Nation Participation in COVID-19 Vaccines
Source: Int J Environ Res Public Health. 2022 Dec 20;20(1):31. doi: 10.3390/ijerph20010031 (PMC9819330; doi:10.3390/ijerph20010031)
Supplement: Supplementary file 1 [file ijerph-20-00031-s001.zip › Handout S9.pdf]

COVID-19 CLINICAL TRIALS

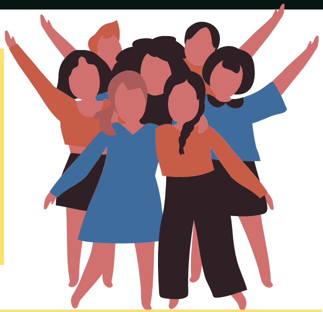

WHAT ARE CLINICAL TRIALS?

A clinical trial is medical research that tests how therapies and treatments work for people.

Clinical research has led to or helped improve every vaccine and treatment available.

WHY SHOULD I VOLUNTEER FOR A CLINICAL TRIAL?

- All clinical research, including research on COVID-19 vaccines and treatments, depends on volunteers.
- Native Americans remain the least represented in research that can prevent or treat COVID-19. Participation in these trials could improve health outcomes for Native Americans.
- Different people can respond to vaccines & treatments in very different ways. That's why there is a need for volunteers to come from every community and racial and ethnic background.

NORTHERN ARIZONA UNIVERSITY
Center for Health Equity Research

Resource: Clinicaltrials.org

Funded By NIH/NIMHD U54MD012388

WHAT ARE THE BENEFITS OF VOLUNTEERING FOR A CLINICAL TRIAL?

- Making a difference in your community and world-wide to stop the COVID-19 pandemic.
- Making sure the vaccines and/or treatments will work for as many people as possible.
- Protecting yourself, your family, and your elders who may be at risk for COVID-19.
- Helping everyone get back to their daily routines.

INDIAN COMMUNITY HEALTH REPRESENTATIVE

GREAT SEAL WHITE MOUNTAIN APACHE TRIBE

Did you know:

Native and Indigenous Researchers are represented at a national level to help with the COVID-19 pandemic?

These researchers come from a variety of tribal/Indigenous communities; like Navajo Nation, Seneca Nation, Eastern Band Cherokee Nation, Alaska Villages, Hawaii, and Choctaw Nation of Oklahoma.

Whiteriver Indian Hospital: (928) 338-5549
